# Carbon footprint dataset of concrete based on field surveys at commercial mixing plants in Shandong, China

**DOI:** 10.1038/s41597-026-06789-0

**Published:** 2026-02-17

**Authors:** Ditao Niu, Juan Zhou, Bingbing Guo

**Affiliations:** 1 Department of School of Civil Engineering, Xi’an University of Architecture and Technology, Xi’an, 710055 China; 2 State Key Laboratory of Green Building in Western China, Xi’an University of Architecture and Technology, Xi’an, 710055 China

**Keywords:** Environmental sciences, Environmental impact

## Abstract

Carbon dioxide (CO_2_) emissions from concrete have grown rapidly, ranking second after the power sector. Current emission factors often overlook regional heterogeneity. To bridge this knowledge gap, this study takes Shandong Province, a typical region in China, as a case study. Considering the difference in geography, history, culture, and economic development, Shandong is divided into five subregions: Eastern, Western, Southern, Northern, and Central Shandong. This study developed a fundamental carbon footprint dataset of concrete by collecting 993 mix proportions of strength grades (C25-C60) from field surveys over the past five years. Statistical analysis showed that raw material dosages followed normal distributions (Kolmogorov-Smirnovtest, p > 0.05), while transportation distances and electricity consumption followed lognormal distributions. Based on statistical characteristics, a Monte Carlo simulation with 10,000 iterations was conducted to establish a stochastic model for carbon emissions accounting. Model performance was validated against survey data, achieving a mean absolute percentage error (MAPE) of 1.89% and a coefficient of determination (R²) of 0.9904. Sensitivity analysis identified cement dosage as the key driver of emissions.

## Background & Summary

Concrete is the second most consumed material globally, surpassed only by water^1,2^. Due to its abundant availability, cost-effectiveness and local accessibility^3^, concrete has emerged as the most extensively utilized construction material worldwide^4^. In 2020, global concrete consumption reached approximately 14 billion cubic meters^5^. However, carbon emissions from concrete production make a significant contribution to both the construction sector and global greenhouse gas emissions, accounting for about 7–9% of total anthropogenic CO_2_ emissions worldwide^6^. Consequently, reducing carbon emissions from concrete production has become a global consensus and shared objective^7^.

The quantification of carbon emissions in concrete production serves as the basis for emission accounting and reduction management^8^. Several general carbon emission datasets have been established, including the Intergovernmental Panel on Climate Change (IPCC) EFDB, the European Environment Agency EFDB the US Environmental Protection Agency EFDB, and the UK Department of Environment EFDB^9^. At present, China’s carbon emission dataset mainly refers to ISO 14044 (GB/T 24044-2008)^10^ and ISO 14040 (GB/T 24040-2008)^11^ and other life cycle assessment (LCA) standards for data accounting^12^, and combines industry-specific specifications such as GB/T 29157-2012^13^ and GB/T 32151-2015^14^ to provide basic data support. However, existing concrete carbon footprint dataset generally lack regional representativeness^8,15,16^, and their reliability and applicability are constrained by multiple factors such as geographical coverage, technical characteristics, accounting standards, and sample size^17,18^. The dataset often only reflect carbon emission levels within their own national contexts and cannot be directly applied to other regions. Therefore, developing region-specific carbon emission dataset for concrete has become an urgent research priority.

**Fig. 1:**
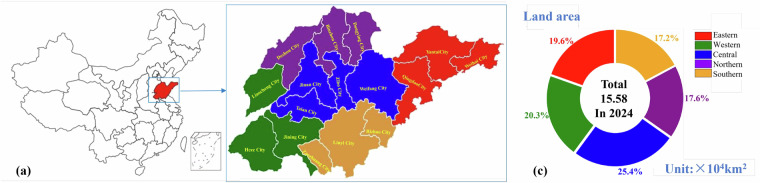
Location and administrative area of Shandong province: (**a**) location of Shandong province in China; (**b**) distribution of cities in Shandong Province; (**c**) regional divisions of Shandong Province.

China, as a major country in infrastructure development, produces more than half of the world’s concrete, and its output continues to grow with ongoing industrialization. According to statistics from the concrete industry^19,20^, China’s concrete output in 2021 reached approximately 3.29 billion cubic meters^21^, an increase of about 210 million cubic meters compared to 2020, representing a year-on-year growth of about 6.9%, ranking first globally^22^. Greenhouse gas emissions generated during its production process account for approximately 8% to 9% of the world’s total emissions^23^. Shandong, as a representative region in China, is selected as a case study for developing a basic carbon-footprint dataset. Based on its geographical features, industrial structure, and patterns of economic development, Shandong is divided into five major subregions: Eastern Shandong, Western Shandong, Southern Shandong, Northern Shandong, and Central Shandong (Fig. 1). As a major industrial province and a key building materials production base, Shandong has consistently ranked among the top three provinces nationwide in terms of industrial added value, with the building materials industry accounting for about 15% of the total^24^. As a major industrial province and key building materials production base, its industrial and energy structure, including reliance on coal and largescale cement and concrete production capacity, closely mirrors national conditions. The province’s industrial system includes heavy chemical industry cities, port-based industrial cities, and resource-based cities, resulting in significant differences in concrete demand across various industrial structures^25–27^. Shandong emitted 936 million tons of carbon in 2020, accounting for about 9% of the country’s total carbon emissions, making it the country’s largest carbon emitter province^28^. However, due to limited data availability, no systematic assessment of the carbon footprints of commercial mixing plants has been conducted across different regions, and a concrete carbon footprint dataset that accounts for regional differences is lacking. Focusing on Shandong allows the construction of a high-quality and scalable dataset that captures regional differences in raw-material supply chains, transportation modes, industrial systems, and production practices, while also reflecting the temporal and spatial variability typical of the national concrete industry. This makes Shandong an ideal and representative region for developing a benchmark concrete carbon-footprint dataset, providing scientific and practical guidance for carbon emission management in China’s concrete sector.

This study developed a foundational dataset for concrete production carbon footprints based on field surveys of 993 concrete mix designs collected from commercial mixing plants in Shandong Province over the past five years. The dataset encompasses detailed data on raw material brands and specifications, transportation distances and methods, as well as energy consumption during concrete mixing. This study employed statistical analysis to identify the distribution characteristics of key factors influencing carbon emissions and established a stochastic carbon accounting model based on 10,000 Monte Carlo simulations. Furthermore, moment-independent sensitivity analysis was applied to quantify the impact of each factor on carbon emissions. Finally, the spatiotemporal characteristics of concrete carbon emissions in Shandong Province were revealed, providing a scientific basis for regional carbon management and the formulation of mitigation strategies.

## Methods

### Boundary definition of carbon emission of concrete

Since 2020, the Shandong Provincial Department of Industry and Information Technology has organized specialized inspection teams for various industries to ensure the integrity and authority of industrial energy conservation supervision^29,30^. Accordingly, the temporal scope of this dataset begins in 2020. This timeframe ensures consistency in data quality and aligns with the period of enhanced industrial regulation in the province.

The spatial coverage of this dataset encompasses the entire Shandong Province, subdivided into five representative regions: Eastern Shandong (Qingdao City, Yantai City, Weihai City), Western Shandong (Jining City, Heze City, Liaocheng City), Central Shandong (Jinan City, Zibo City, Tai’an City, Weifang City), Northern Shandong (Dezhou City, Binzhou City, Dongying City), Southern Shandong (Rizhao City, Linyi City, Zaozhuang City). To ensure the representativeness of the dataset, a stratified sampling strategy was employed to select concrete batching plants across Shandong Province. Within each region, samples were chosen based on plant size and operation type, while also considering survey accessibility to ensure the feasibility of field investigations. This approach effectively captures spatial variability in regional distribution, plant scale, energy structure, and industrial characteristics, while also reflecting significant variations in concrete carbon footprint and production capacity. In total, 993 concrete mix proportion datasets were collected from commercial batching plants across these regions, providing comprehensive coverage of the provincial concrete production system.

**Fig. 2:**
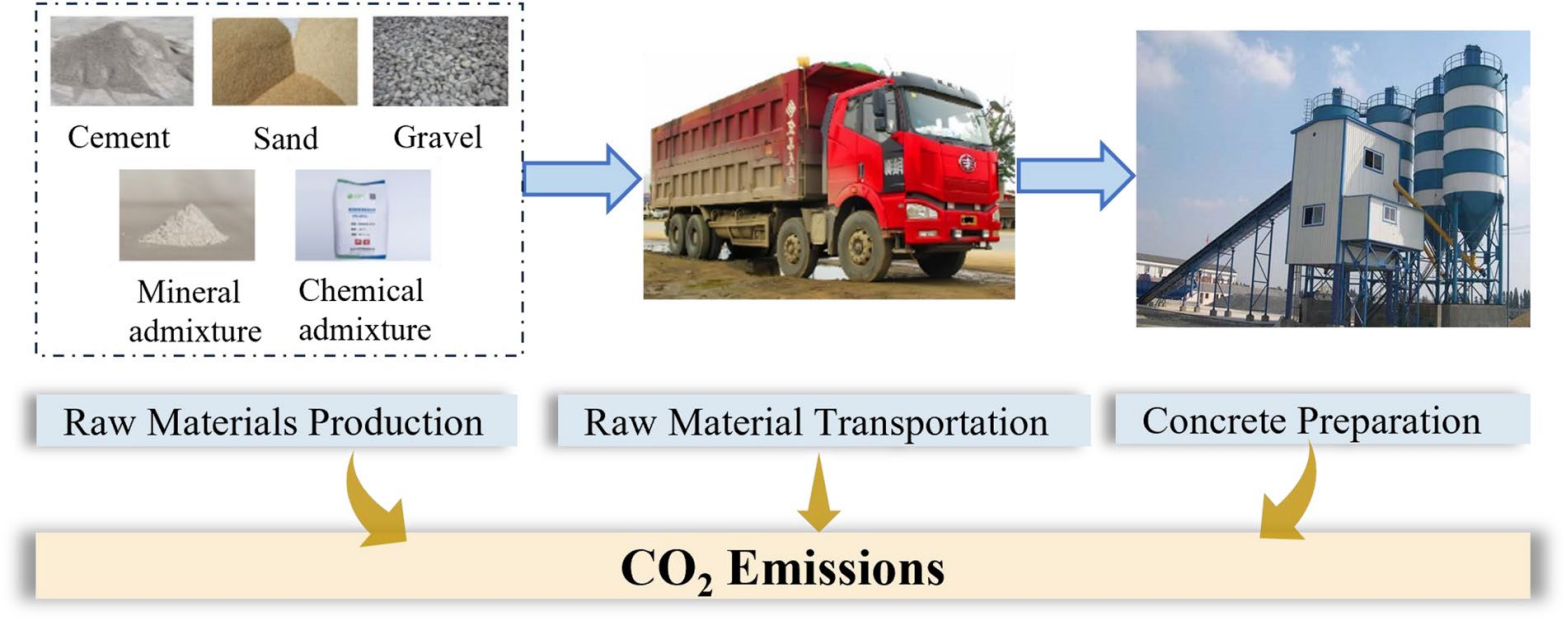
Construction flows of CO_2_ emission inventory of concrete.

The concrete counting boundary for concrete production was defined using a “cradle-to-gate” approach^31^, as shown in Fig. 2, encompassing carbon emissions from three key stages: (1) raw material production, (2) raw material transportation, and (3) concrete preparation^32^. This boundary captures the majority of emissions generated during concrete production while maintaining a manageable scope for analysis. The functional unit was defined as 1m^3^ of concrete, ensuring standardized comparisons across different strength grades and production scenarios^33^.

### Data collection and processing

The primary annual plant-level data, beginning in 2020, were collected primarily through field surveys, interviews with technical staff and enterprise managers, and production and energy consumption reports provided by the enterprises. To ensure representativeness across Shandong Province, a stratified sampling strategy was employed: the province was divided into five subregions, and within each subregion, concrete mixing plants were selected based on production scale, plant type, and accessibility. The dataset includes993sets of concrete mix proportions for grades C25-C60, along with detailed information on raw materials (brand, origin, manufacturer, production date, type, and grading), as well as transportation methods and distances of raw materials and energy consumption during production. Data collection covered the period from 2020 to 2024, and rigorous measures were taken to ensure accuracy and consistency, including cross-validation of reported values, verification of plant records, and documentation of temporal changes in production processes, energy sources, and transportation modes. This comprehensive approach ensures that the dataset is representative, reliable, and suitable for carbon footprint assessment and related analyses.

A systematic data preprocessing workflow is essential to ensure accuracy and analytical reliability. The procedure includes: cleaning raw data by removing missing, inconsistent, or outlier entries; standardizing and harmonizing records from different mixing plants to eliminate bias caused by varied reporting formats; normalizing and discretizing continuous variables to enhance the robustness of subsequent statistical analyses; performing feature selection and eliminating redundant variables to retain only the most influential parameters for carbon footprint accounting and statistical characterization; and organizing the dataset by temporal and spatial dimensions to enable the investigation of spatiotemporal characteristics in concrete carbon emissions.

**Fig. 3:**
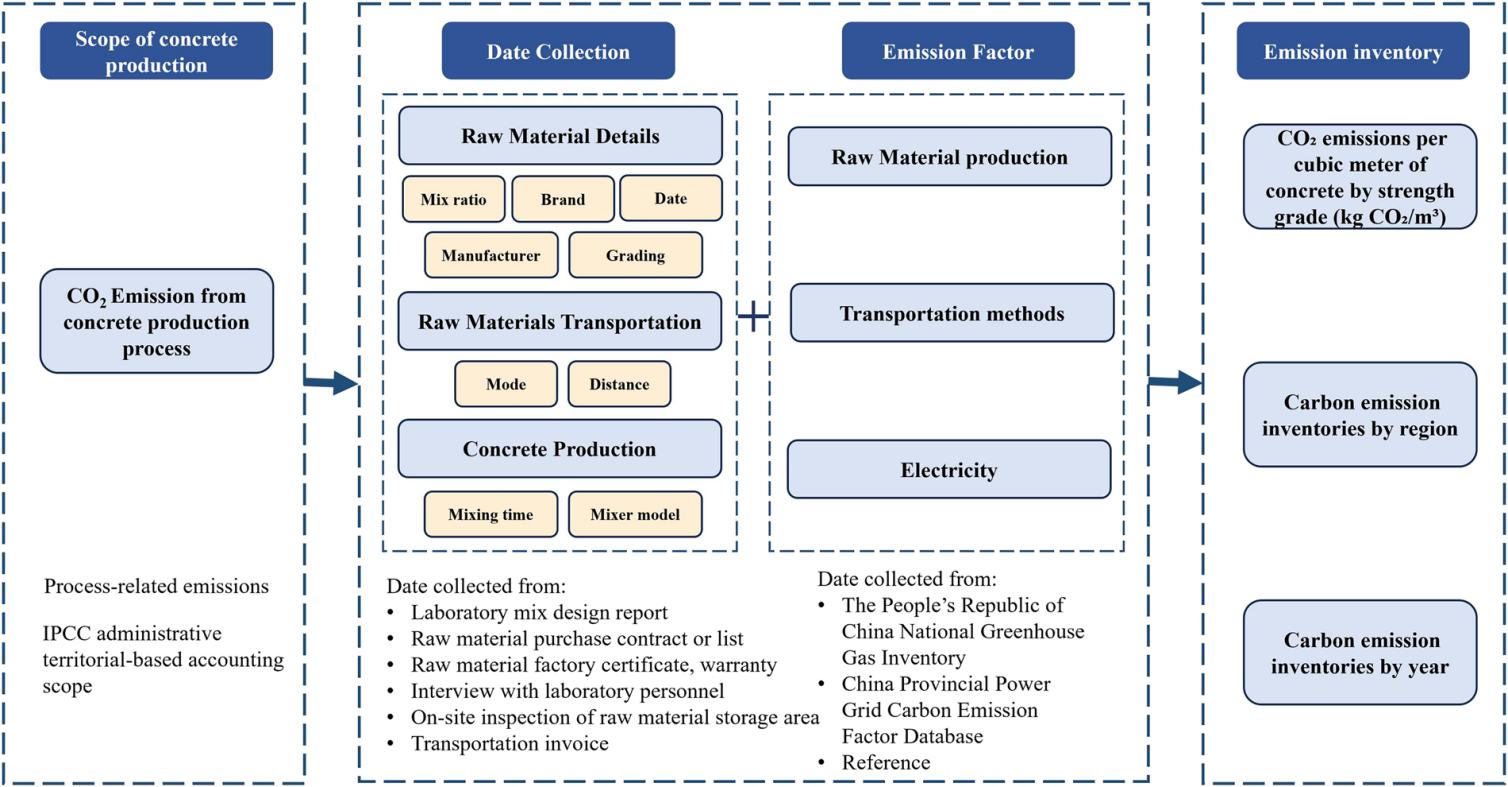
Construction flows of Shandong’s provincial CO_2_ emission inventory from the concrete production process.

The energy intensity and CO_2_ emissions factors are regarded as secondary data, are generally calculated from the primary data followed by the Standard for Calculation of Building Carbon Emissions (GBT 51366-2019)^34^, and 2006 IPCC Guidelines for National Greenhouse Gas Inventories^35^. The entire processing work of the present dataset is described in Fig. 3.

### Accounting process-related CO_2_ emissions of concrete and emission coefficient

All carbon emission factors used in this study are listed in Tables 1, 2. Although we surveyed the cement brands used in each mixing plant, collected samples, and conducted XRF analyses to estimate brand-specific emission factors, the complexity of upstream cement production led us to adopt a unified emission factor from existing literature in this study. The carbon emission factors for other raw materials used in concrete, as well as the emission factors for transportation, were based on the National Materials Science Data Sharing Platform^36^, the Construction Carbon Emission Calculation Standard (GB/T 51366-2019)^34^ and the research of other scholars^37,38^. In addition, Indirect emissions from electricity use during concrete production are calculated using the 2021 national, regional, and provincial average carbon dioxide emission factors for electricity, which is 0.6838 CO_2_e/kWh for Shandong^39^. The specific calculation formula is as follows:

**Table 1:** Carbon dioxide emissions per unit of raw material production process.

Number	Raw Material Type	Unit	Carbon Dioxide Emissions per unit of Raw Material Production Process (kgCO_2_/kg)
1	cement (P O42.5)	kg	0.785
2	mineral powder	kg	0.06235
3	fly ash	kg	0.0345
4	natural sand	kg	0.003984
5	manufactured sand	kg	0.0417
6	gravel	kg	0.00218
7	water	kg	0.000168
8	chemical admixture (polycarboxylic acid)	kg	1.064

**Table 2:** Carbon dioxide emission factor for unit raw material transportation mode.

Number	Raw Material Transportation Mode	Unit	Carbon dioxide Emissions per unit of Raw Material Production Process (kgCO_2_/kg)
1	Light gasoline truck transportation (2t capacity)	km	0.334
2	Medium-sized gasoline truck transportation (8t capacity)	km	0.115
3	Heavy-duty gasoline truck transportation (10t capacity)	km	0.104
4	Heavy-duty gasoline truck transportation (18 capacity)	km	0.104
5	Light diesel truck transportation (2t capacity)	km	0.286
6	Medium-sized diesel truck transportation (8t capacity)	km	0.179
7	Heavy-duty diesel truck transportation (10t capacity)	km	0.162
8	Heavy-duty diesel truck transportation (18t capacity)	km	0.129
9	Heavy-duty diesel truck transportation (30t capacity)	km	0.078
10	Heavy-duty diesel truck transportation (46t capacity)	km	0.057
11	Rail transportation (average in the Chinese market)	km	0.01

The total CO_2_ emissions per cubic meter of concrete are calculated as:

1 $${E}_{{\rm{total}}}={E}_{{\rm{m}}}+{E}_{{\rm{t}}}+{E}_{{\rm{c}}}$$

where:

*E*_m_ represents the emissions from raw material production (kg CO_2_e/m^3^),

*E*_t_ represents the emissions from raw material transportation (kg CO_2_e/m^3^),

*E*_*c*_ represents the emissions from concrete production (kg CO_2_e/m^3^).

*E*_m_ includes the carbon emissions associated with the extraction, processing, and manufacturing of raw materials such as cement, water, sand, gravel, fly ash, mineral powder, and chemical admixtures.

2 $${E}_{m}=\mathop{\sum }\limits_{i}^{n}{Q}_{i}\times E{F}_{i}$$

where:

*Q*_*i*_ represents the quantity of raw material i used per cubic meter of concrete (kg/m^3^),

*EF*_*i*_ represents the emission factor of raw material i (kg CO_2_e/kg).

*E*_*t*_ accounts for emissions generated from transporting raw materials from suppliers to concrete plants. The emissions depend on factors such as transportation distance, mode (e.g., truck, rail, or ship), and fuel consumption.

3 $${E}_{t}=\mathop{\sum }\limits_{i}^{n}{Q}_{i}\times {D}_{i}\times E{F}_{t}$$

where:

*D*_*i*_ represents the transportation distance for raw material (km),

*EF*_*t*_ represents the emission factor for transportation [kg CO_2_e/(kg·km)].

*E*_*c*_ includes emissions from the energy consumption of concrete mixing equipment, such as electricity use in batching and mixing operations.

4 $${E}_{{\rm{c}}}=C\times E{F}_{{\rm{power}}}$$

where:

*E*_*c*_ represents the carbon emissions from the concrete production stage (kg CO_2_e/m^3^),

*C* represents the electricity consumption during concrete production (kWh/m^3^),

*EF*_power_ represents the carbon emission factor of electricity in Shandong Province (kg CO_2_e/kWh).

## Data Records

The dataset consists of five Excel files, each corresponding to one of the five regions of Shandong Province: Eastern, Western, Southern, Northern, and Central Shandong. Each data file encompasses the following detailed information for concrete grades C25 to C60:

1. Mix Design: Precise mix proportions.
2. Raw Materials: Specifications including brand, manufacturer, and classification.
3. Logistics: Transportation methods and distances.
4. production Process: Specifications of the mixing equipment and mixing duration.

This peer-reviewed version corresponds to the static dataset submitted to the Science Data Bank website. It is accessible through a link: 10.57760/sciencedb.28528^40^.

## Data Overview Statistical analysis of raw material dosages

**Fig. 4:**
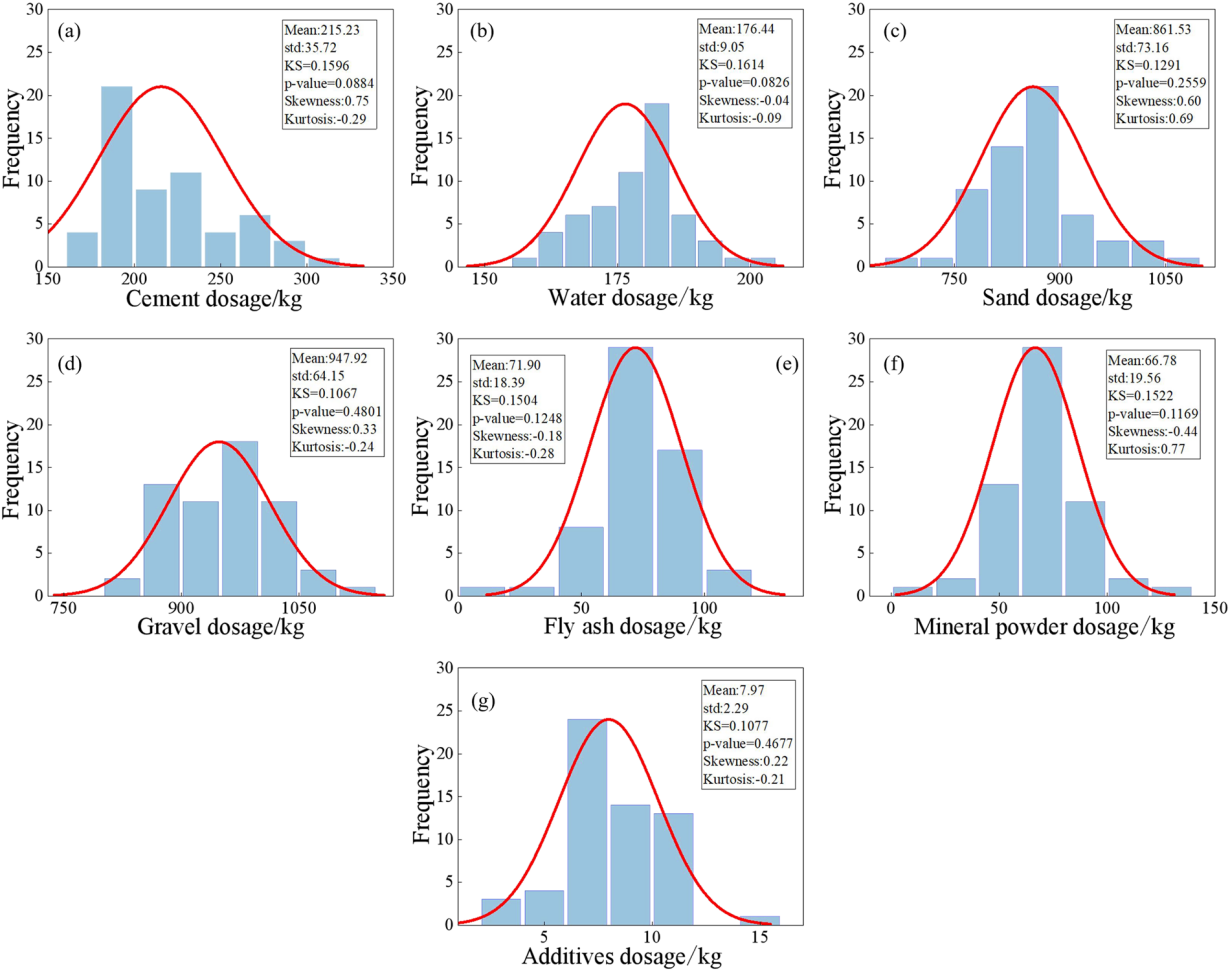
Statistical characteristics of raw material dosages of C25 concrete.

**Fig. 5:**
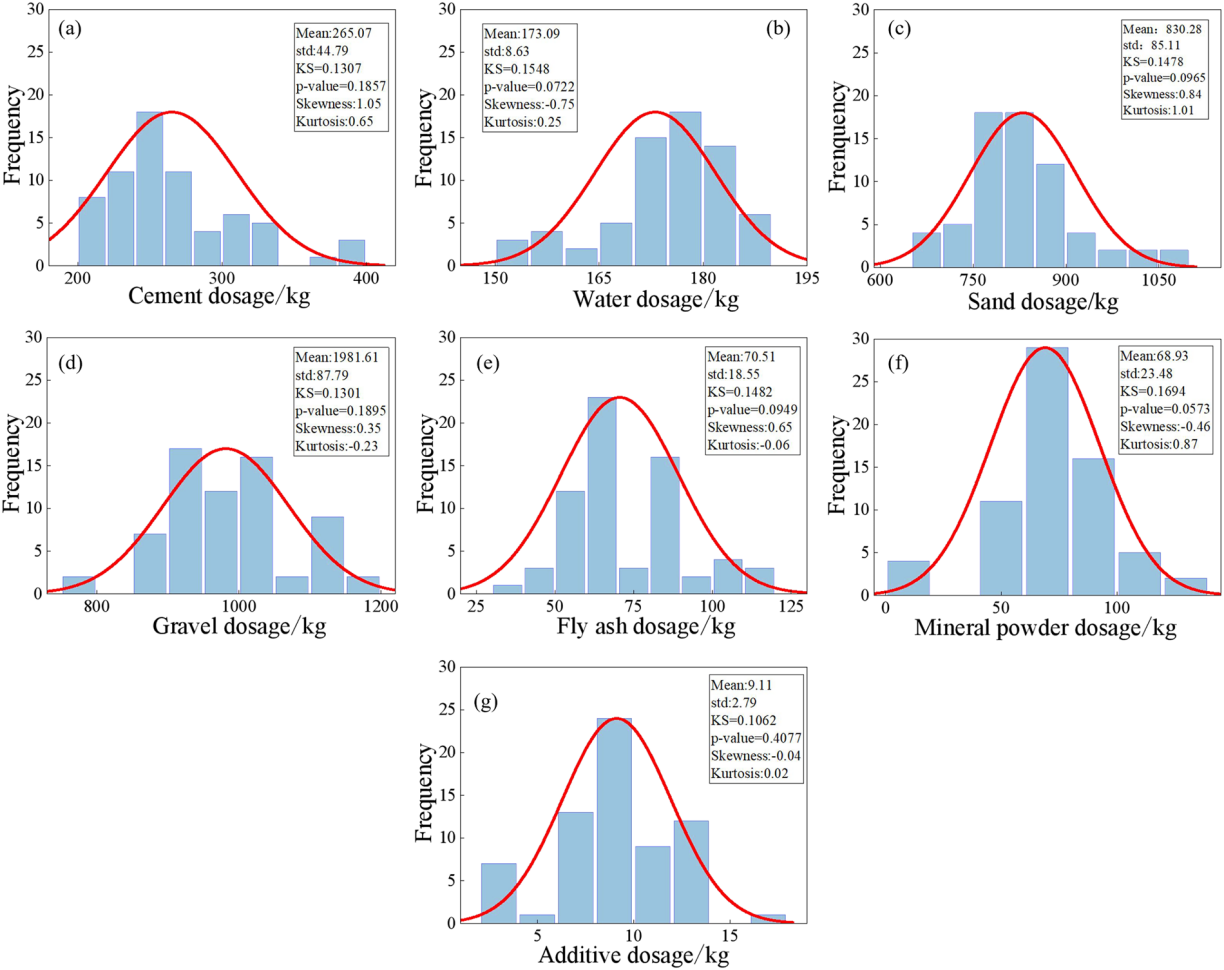
Statistical characteristics of raw material dosages of C30 concrete.

**Fig. 6:**
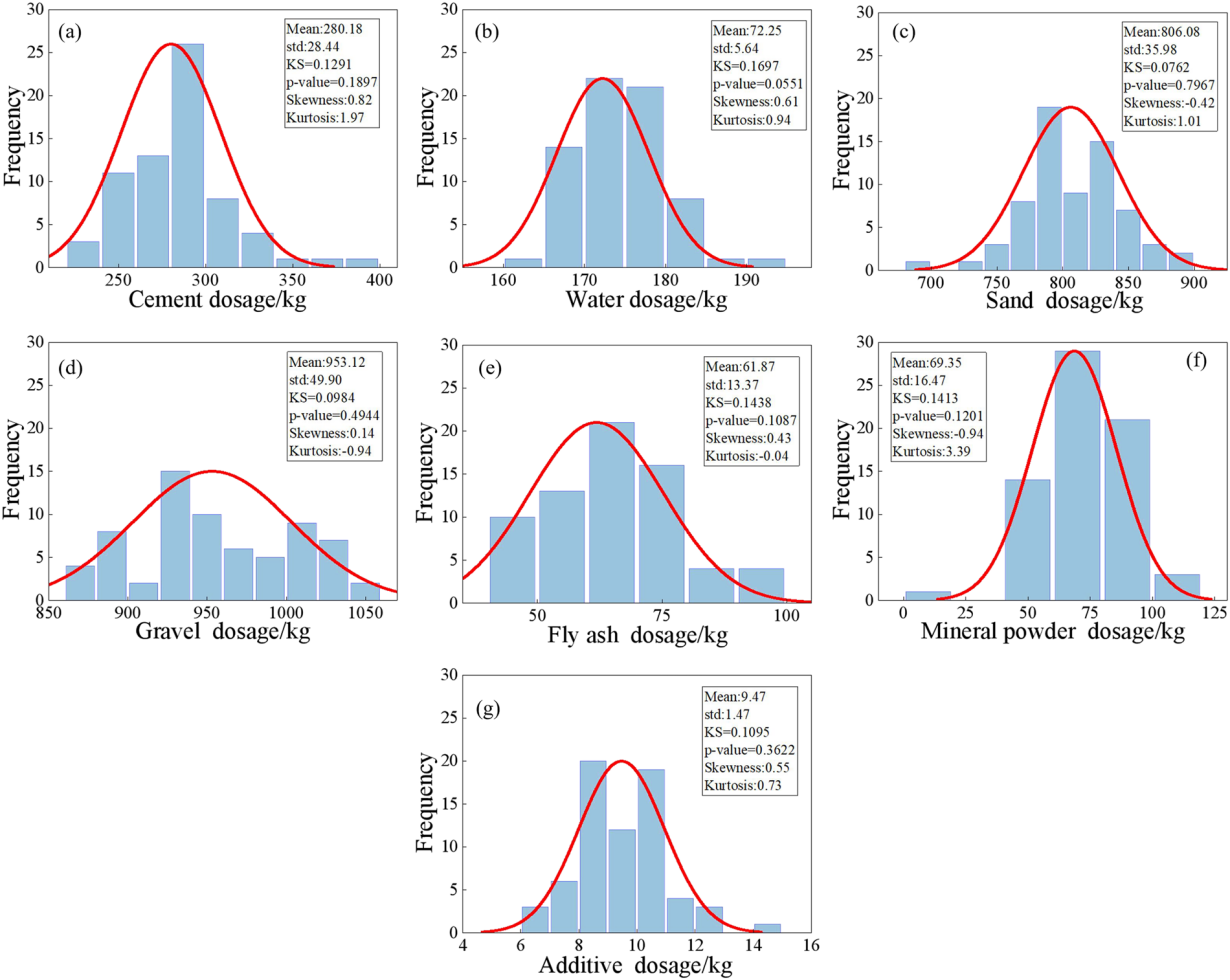
Statistical characteristics of raw material dosages of C35 concrete.

**Fig. 7:**
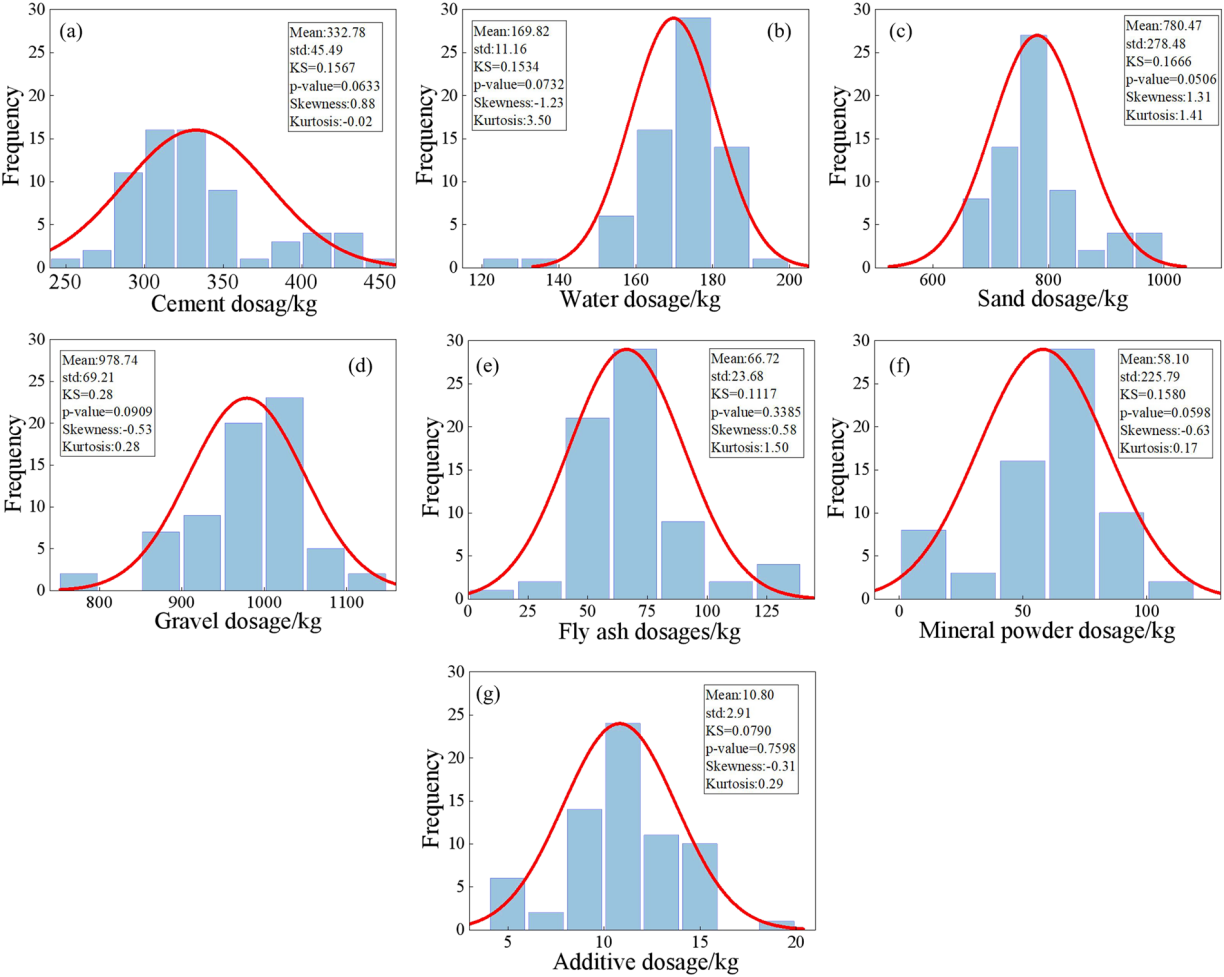
Statistical characteristics of raw material dosages of C40 concrete.

**Fig. 8:**
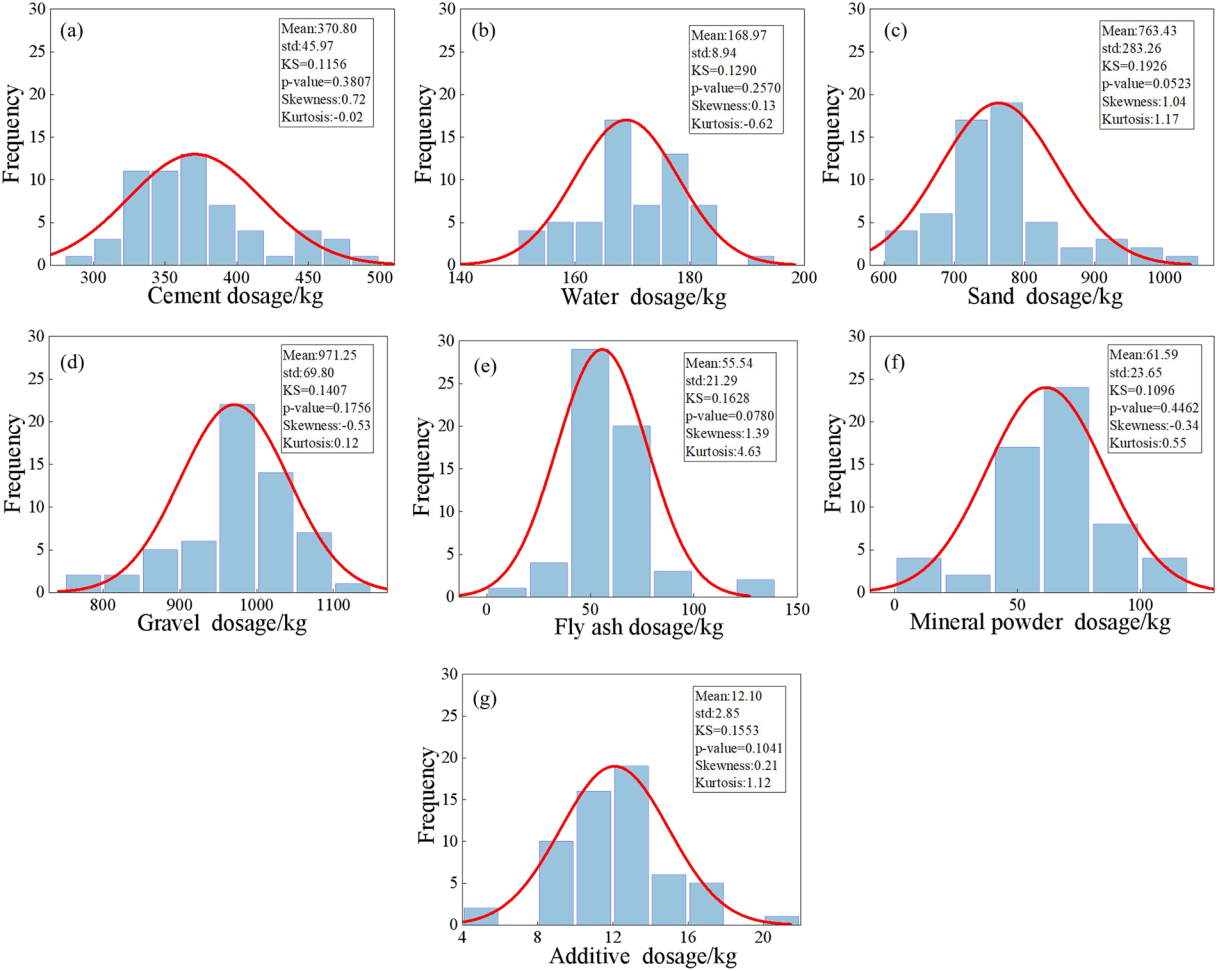
Statistical characteristics of raw material dosages of C45 concrete.

**Fig. 9:**
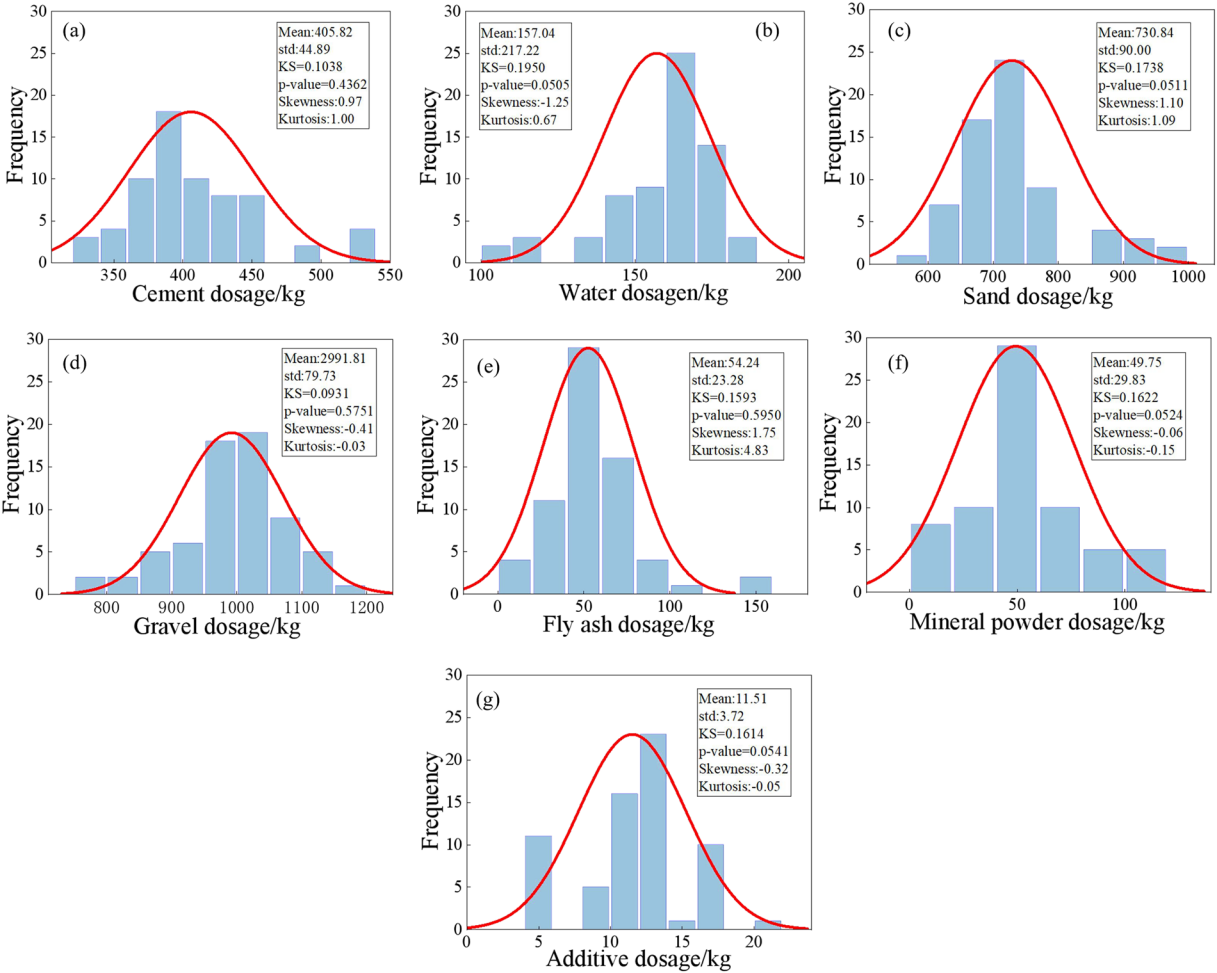
Statistical characteristics of raw material dosages of C50 concrete.

**Fig. 10:**
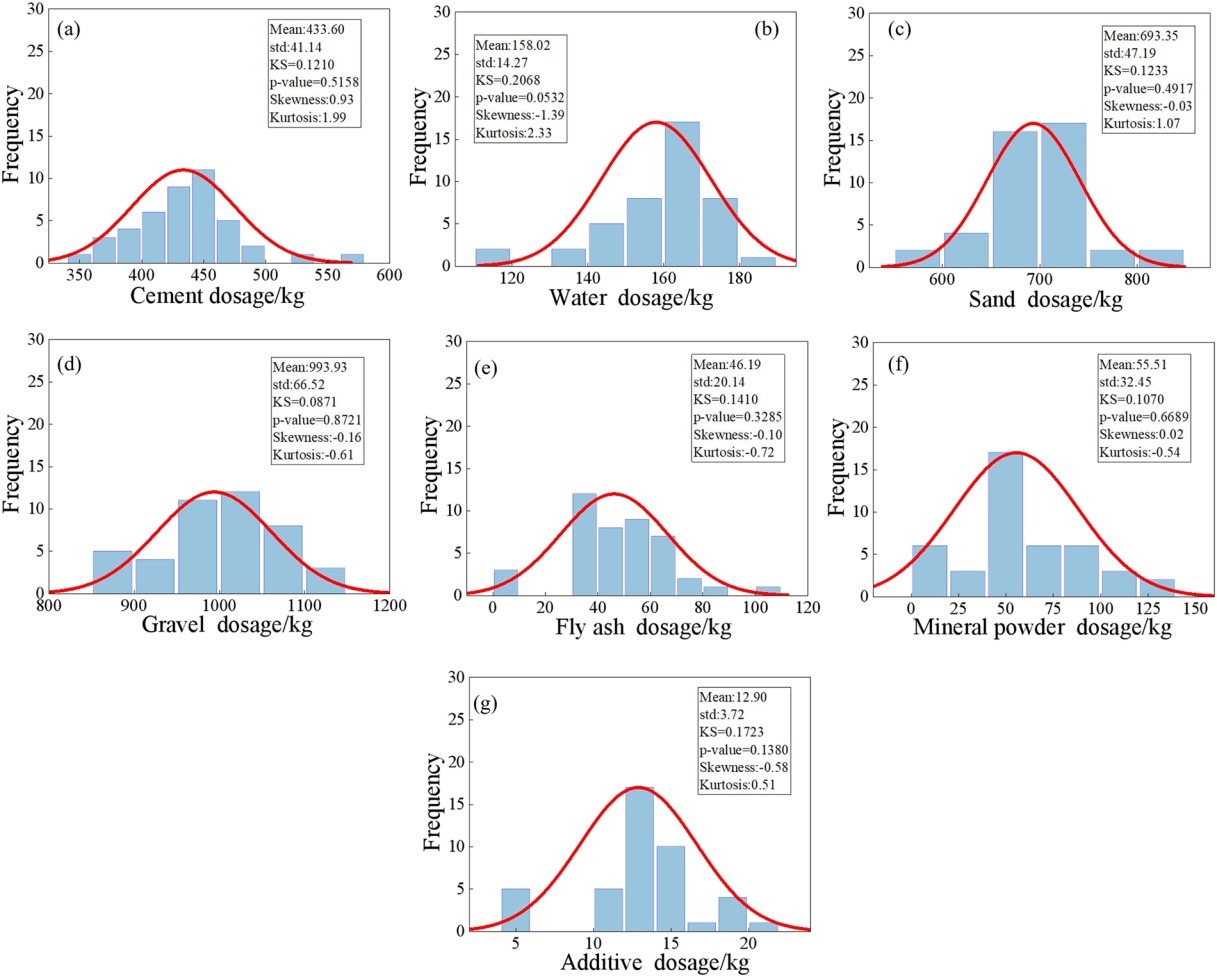
Statistical characteristics of raw material dosages of C55 concrete.

**Fig. 11:**
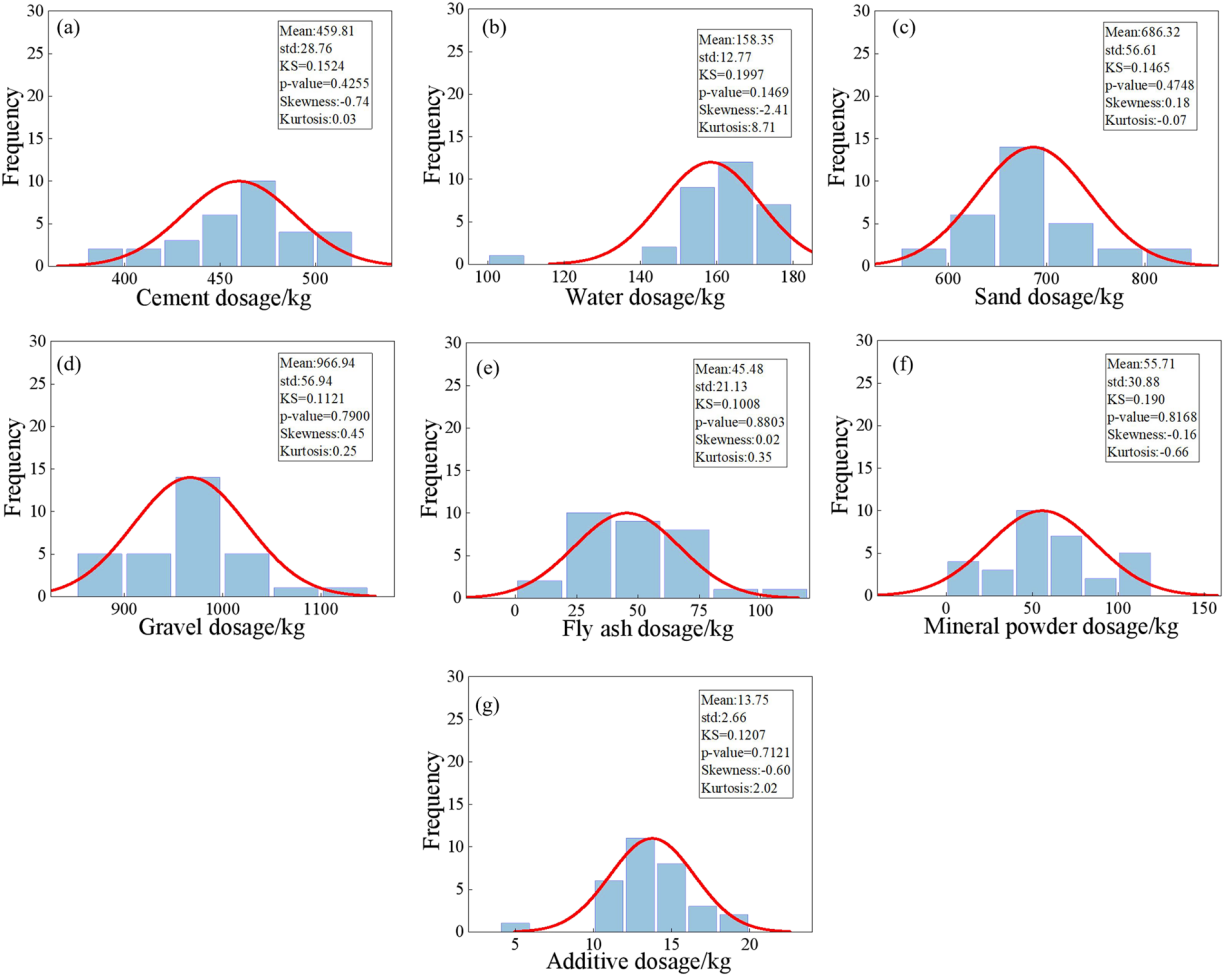
Statistical characteristics of raw material dosages of C60 concrete.

To verify whether the quantities of raw materials in concrete follow a normal distribution, the Kolmogorov-Smirnov (KS) test was conducted^41,42^. The Kolmogorov-Smirnov test results indicated that all p-values were greater than 0.05, suggesting that the raw material dosages for concrete grades C25 to C60 follow a normal distribution, as illustrated in Figs. 4–11. Taking C25 grade concrete as an example, Fig. 4 presents a systematic analysis of the statistical characteristics of seven raw materials, including their probability distributions, mean values, standard deviations, and KS test results. The results indicate that the skewness of most materials is close to zero, suggesting a relatively symmetrical distribution. Moreover, the kurtosis values are generally greater than 3, implying that the data are highly concentrated around the mean. In addition, the standard deviations are relatively small, further demonstrating that the variability in the mix proportion design is low. Overall, these results confirm that the raw material dosages meet the stability and consistency requirements of engineering design^43^.

**Fig. 12:**
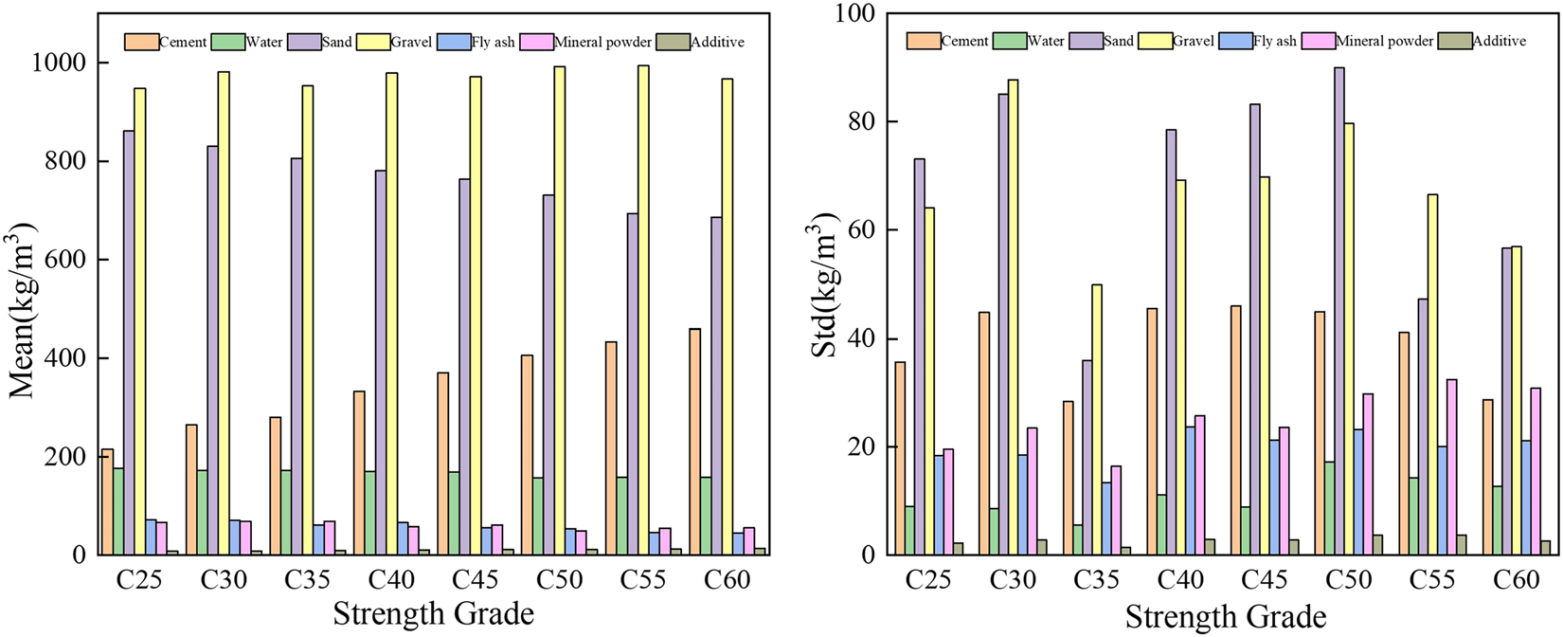
The mean and standard deviation of raw material dosages.

The statistical characteristics of raw materials (mean and standard deviation) across concrete strength grades are illustrated in Fig. 12. As the increases of concrete strength grade, the cement dosages increased significantly from 215.23 kg to 459.81 kg. Water usage varies little among different strength grades, slightly decreases between C50 and C60, and fluctuates mainly between 157 and 176 kg. The sand dosages gradually decrease with increasing of strength grade, with a more pronounced reduction at higher grades. The dosages of gravel fluctuate between 947 and 993 kg and shows a slight increase as strength grade rises. The consumption of fly ash decreases with increasing strength grade, while the content of mineral powder remains relatively stable. In contrast, the dosage of chemical admixtures rises from 7.97 kg to 13.75 kg.

### Statistical analysis of transportation distance of raw materials

**Fig. 13:**
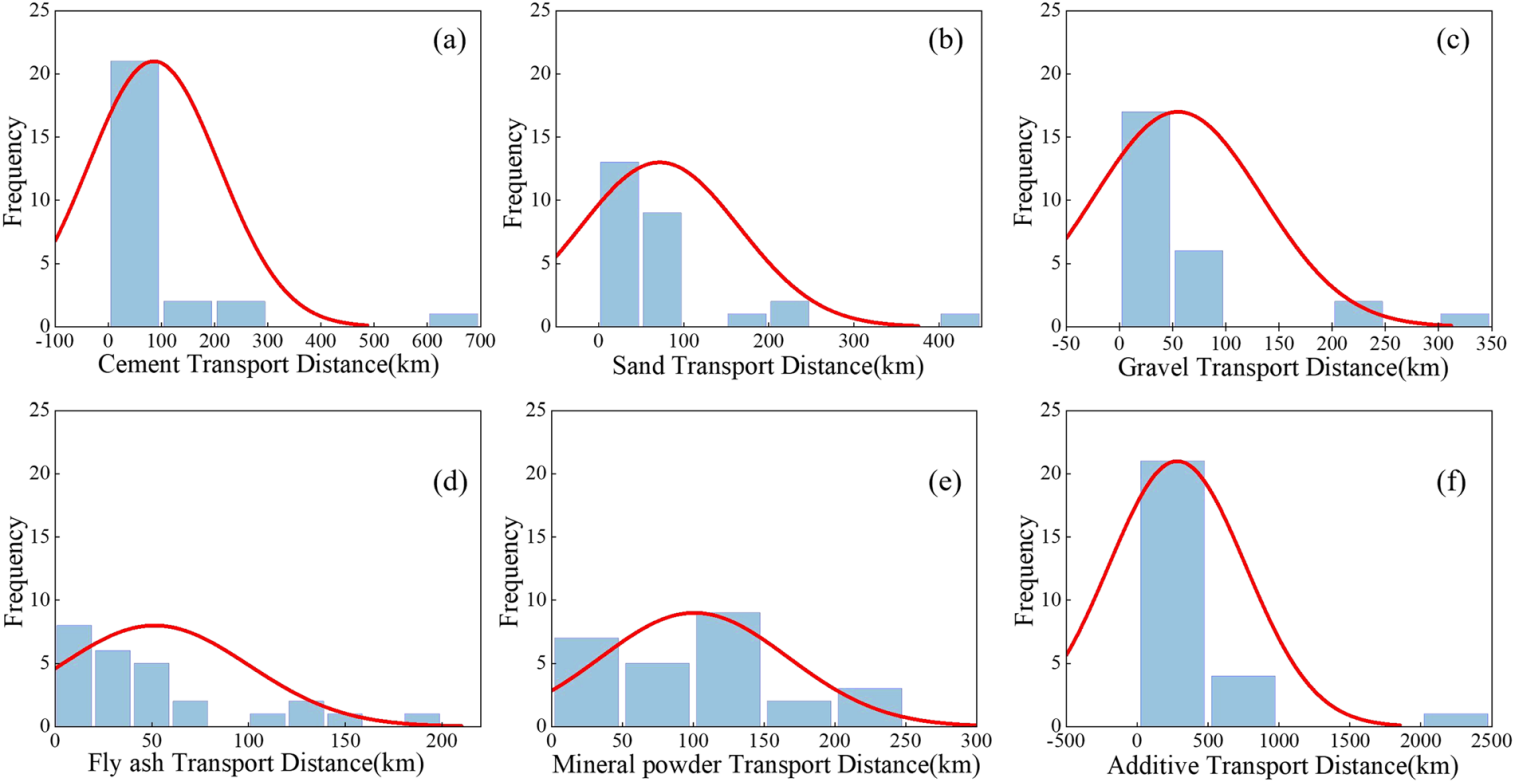
Statistical characteristics of transportation distances for concrete raw materials.

As shown in Fig. 13, the transportation distance of concrete raw materials follows a typical log-normal distribution, showing a right-skewed long-tail feature on the original scale^44^. The results show that the supply mode of concrete raw materials is mainly local procurement (normal distribution core), and long-distance procurement accounts for a small proportion (exponential tail), which conforms to the supply mode of “short-distance transportation as the main and long-distance transportation as the auxiliary”.

### Statistical analysis of transportation methods of raw materials

**Fig. 14:**
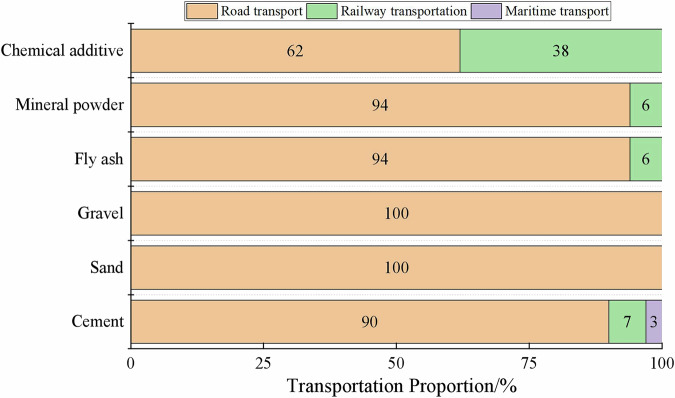
Proportion of different transportation methods for raw materials.

Based on the statistical analysis of the transportation methods of raw materials in Fig. 14, it can be observed that the powdered materials such as cement, fly ash, mineral powder and chemical admixtures are mainly transported by rail and road, while aggregates (sand and gravel) rely entirely on road transportation.

### Statistical analysis of electricity consumption

**Fig. 15:**
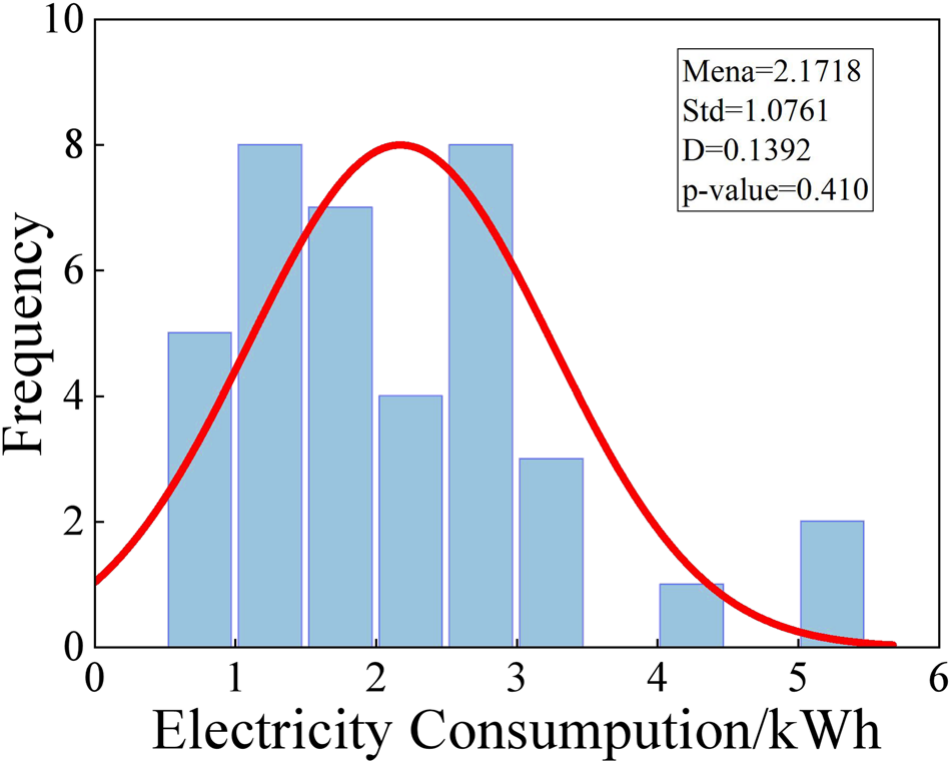
Statistical characteristics of electricity consumption in concrete production.

Based on the field survey data, the unit electricity consumption (kWh/m^3^) in the concrete production process presents a log-normal distribution, as shown in Fig. 15. The statistical parameters show that the mean value (μ = 2.1718) represents the electricity consumption level of the concrete industry in Shandong Province, while the variance (σ^2^ = 1.0761) reveals that there are significant differences in process control among different ready-mixed concrete plants. The KS test results (D = 0.1392, p = 0.410 > 0.05) confirm the validity of the distribution assumption. In addition, about 18.3% of the samples have abnormally high electricity consumption (>3.5 kWh/m^3^).

### Statistical analysis of concrete carbon emissions

**Fig. 16:**
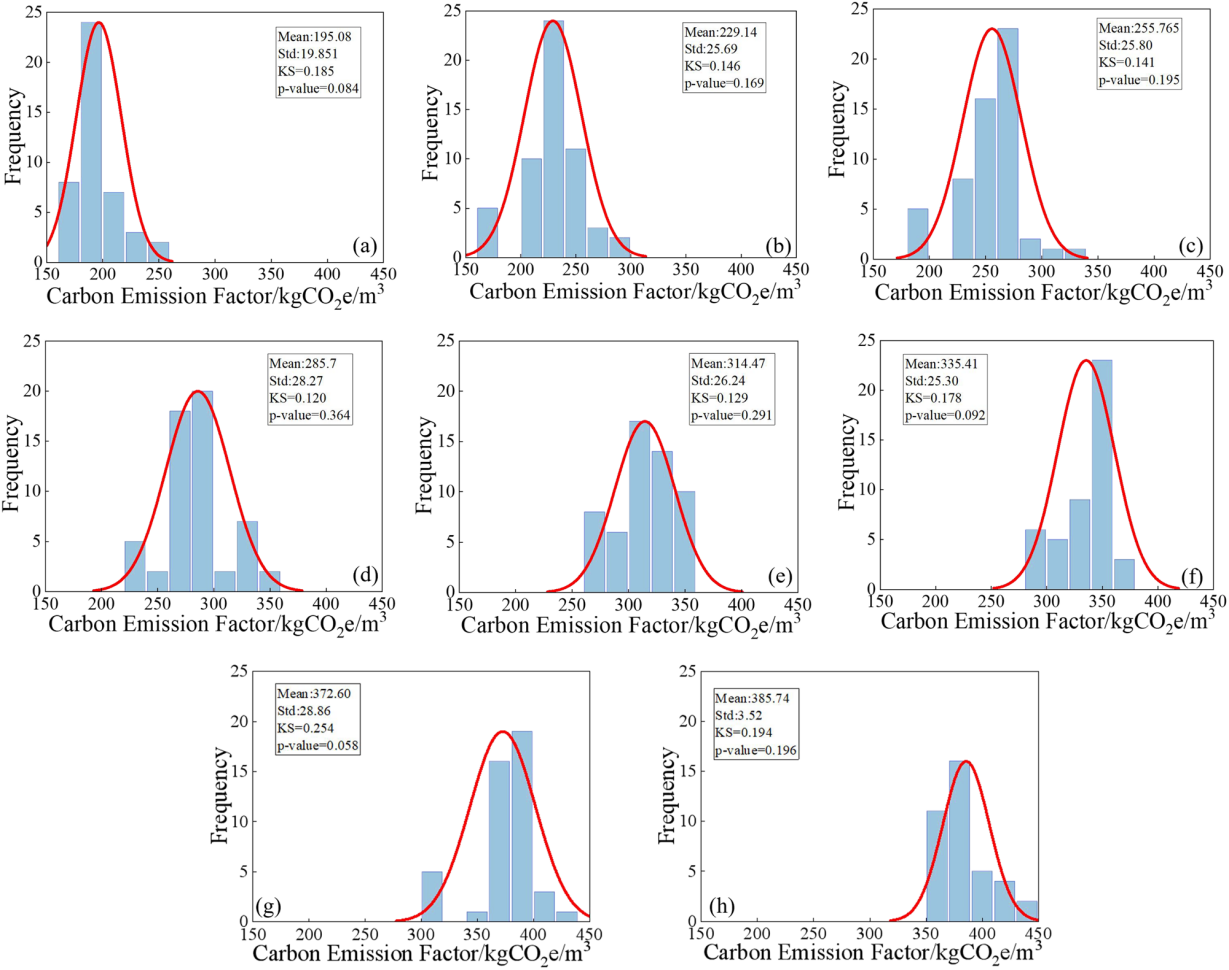
Statistical characteristics of Concrete Carbon Emissions: (a)-(h) represents concrete strength grade C25-C60.

Based on the above methods and fundamental data, and the statistical characteristics of different strength grades are presented in Fig. 16. The results show that the carbon emissions of all strength grades follow a normal distribution (KS test, p > 0.05), with no significant outliers detected. Further analysis reveals a strong linear increase in carbon emissions with increasing strength grade (R² = 0.93), which is consistent with theoretical expectations driven by the higher dosage of cementitious materials. Specifically, the carbon emissions of C60 concrete are 97.97% higher than those of C25 concrete. In addition, the standard deviations of carbon emissions for high-strength concretes (C50~C60) are considerably larger than those for low-strength concretes (C25~C40), reflecting greater variability in mix proportion design and process control during the production of high-strength concrete.

## Technical Validation

### Statistical characteristic analysis and carbon mitigation insights

A systematic analysis of raw material consumption indicates that their usage exhibits a notably stable and consistent distribution throughout the range of C25-C60 concrete strength grades. As concrete strength increases, cement usage rises to meet structural requirements, while sand, aggregate, and water are adjusted to maintain concrete performance. Fly ash usage decreases with higher strength due to its low reactivity, and the content of mineral powder remains relatively stable, influenced by variations in slag reactivity. Chemical admixtures increase to improve workability and strength. The standard deviations of raw materials show little change, likely due to the diversity of mix designs, differences in material properties, and environmental factors. Comparison with national (GB/T 51366-2019, GB/T 50640-2023)^34,45^ and international datasets (IPCC EFDB, Ecoinvent CEM III/B)^35^ confirms that the observed patterns and values are consistent with typical ranges, supporting the reliability and representativeness of the dataset.

The choice of transportation method for concrete raw materials is influenced by material physical properties, logistics economics, and industrial layout. Powdered materials, such as cement, fly ash, mineral powder, and chemical admixtures, have high bulk density and are typically produced at centralized, large-scale facilities. As a result, rail transport is more cost-effective for long-distance, high-volume shipments, particularly when the distance exceeds 350 kilometers^46^, with the carbon emission factor of rail transport being only 0.01 kg CO_2_/(t·km) according to the Chinese standard Building Carbon Emission Calculation Standard (GB/T 51366-2019)^34^. Aggregates, on the other hand, are usually sourced from geographically dispersed quarries near construction sites, making road transport more practical and flexible, although road transport emissions are significantly higher, ranging from 0.057 to 0.334 kg CO_2_/(t·km). Areas close to ports or rail lines facilitate multimodal transport for powdered materials, while regions lacking such infrastructure rely solely on trucks. Most raw material transportation distances follow a log-normal distribution, with local procurement dominating and long-distance shipments forming a long tail. Increasing the share of rail or water transport for long-distance deliveries, optimizing delivery routes, and improving load efficiency to reduce empty runs are effective strategies to lower carbon emissions during the transportation stage.

The extreme electricity consumption values observed in some plants are attributed primarily to aging equipment, particularly mixing stations that have been in service for over a decade, which results in a significantly higher electricity consumption per unit of concrete produced. Compared with the advanced benchmark of 1.8 kWh/m^3^ recommended by the Green Ready-Mixed Concrete Evaluation Standard (GB/T 50640-2023)^45^, the mean value is 21% higher, indicating that there is great potential for energy efficiency improvement in the entire industry. Taking the industry advanced benchmark of 1.8 kWh/m^3^ as the reference, the excess electricity consumption due to aging equipment (e.g., 3.5 kWh/m^3^) leads to an additional 1.7 kWh/m^3^, corresponding to approximately 1.16 kg CO_2_e/m^3^ of extra emissions. Reducing carbon emissions from concrete mixing requires addressing equipment efficiency. Aging plants increase electricity use and CO_2_ emissions, which can be mitigated through predictive maintenance, performance monitoring, and retrofitting with energy-efficient equipment. Complementary policies such as efficiency standards, fiscal incentives, and pilot programs with digital process control can promote adoption across the industry.

**Fig. 17:**
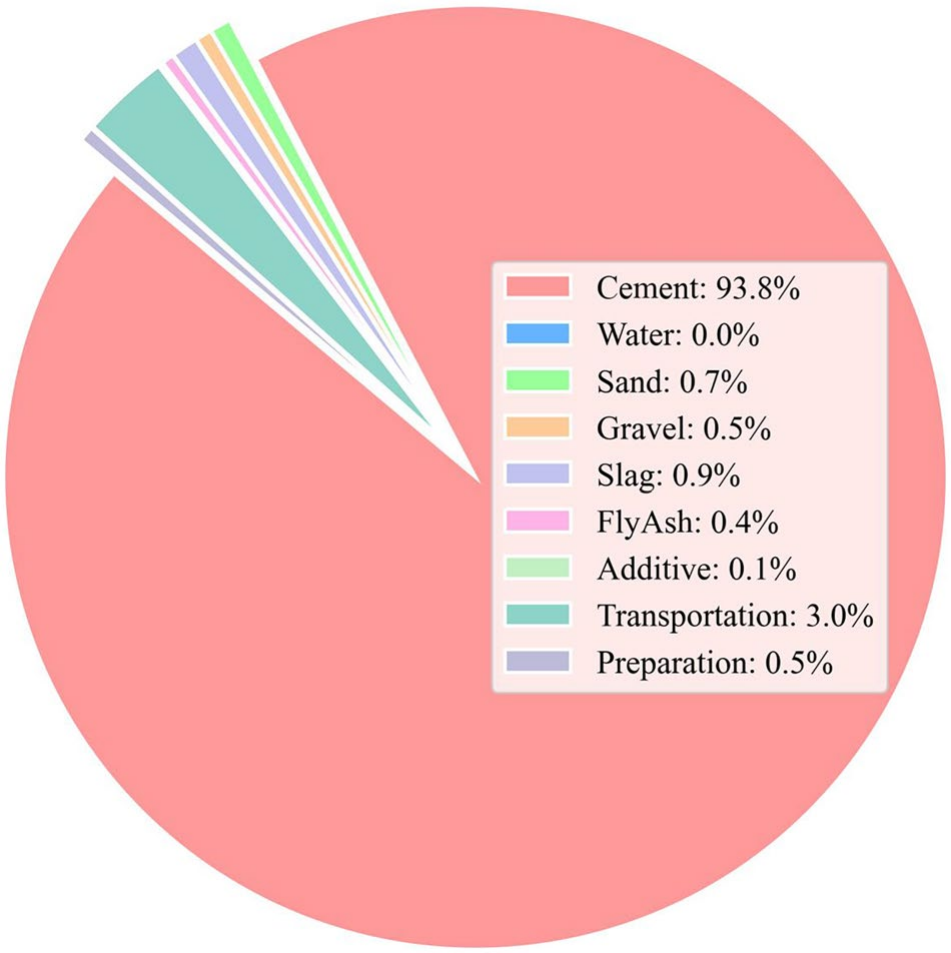
Contribution of different components to the carbon footprint of concrete.

As shown in Fig. 17, cement is the dominant contributor to the carbon footprint of concrete, accounting for 361.13 kg CO_2_e per cubic meter, or 93.8% of total emissions. This decisive role is mainly due to the complex cement production process, which generates carbon emissions from two primary sources: the decomposition of limestone (CaCO_3_) into calcium oxide during calcination (process emissions) and the combustion of fossil fuels to provide the high temperatures required in cement kilns (energy-related emissions). In comparison, other components such as transportation (3.0%), slag, sand, gravel, fly ash, additives, water, and preparation each contribute less than 1% to the total carbon footprint. These results indicate that optimizing cement usage and its supply chain logistics is crucial for reducing the overall carbon emissions of concrete. These results indicate that optimizing cement usage and its supply chain logistics is crucial for reducing the overall carbon footprint of concrete. Based on this finding, several actionable policy and mitigation strategies are proposed: promoting the use of alternative binders (such as fly ash, slag, or other supplementary cementitious materials), improving cement production efficiency standards, and encouraging low-carbon transportation modes can effectively reduce emissions. In addition, concrete producers can optimize mix designs to minimize cement content while maintaining structural performance and improve logistics planning to lower transportation-related emissions. Together, these measures provide a scientific basis for regional carbon management and the formulation of targeted emission reduction strategies.

Research shows that partially replacing cement with supplementary cementitious materials, such as fly ash or slag, can significantly reduce CO_2_ emissions without compromising strength. Blended cements with SCMs and optimized clinker content can cut emissions by 20–40%. Based on these findings, mitigation strategies include promoting alternative binders, optimizing mix designs to minimize cement content while ensuring structural performance, improving production efficiency, and adopting low-carbon transportation.

### Spatial and temporal characteristics

**Fig. 18:**
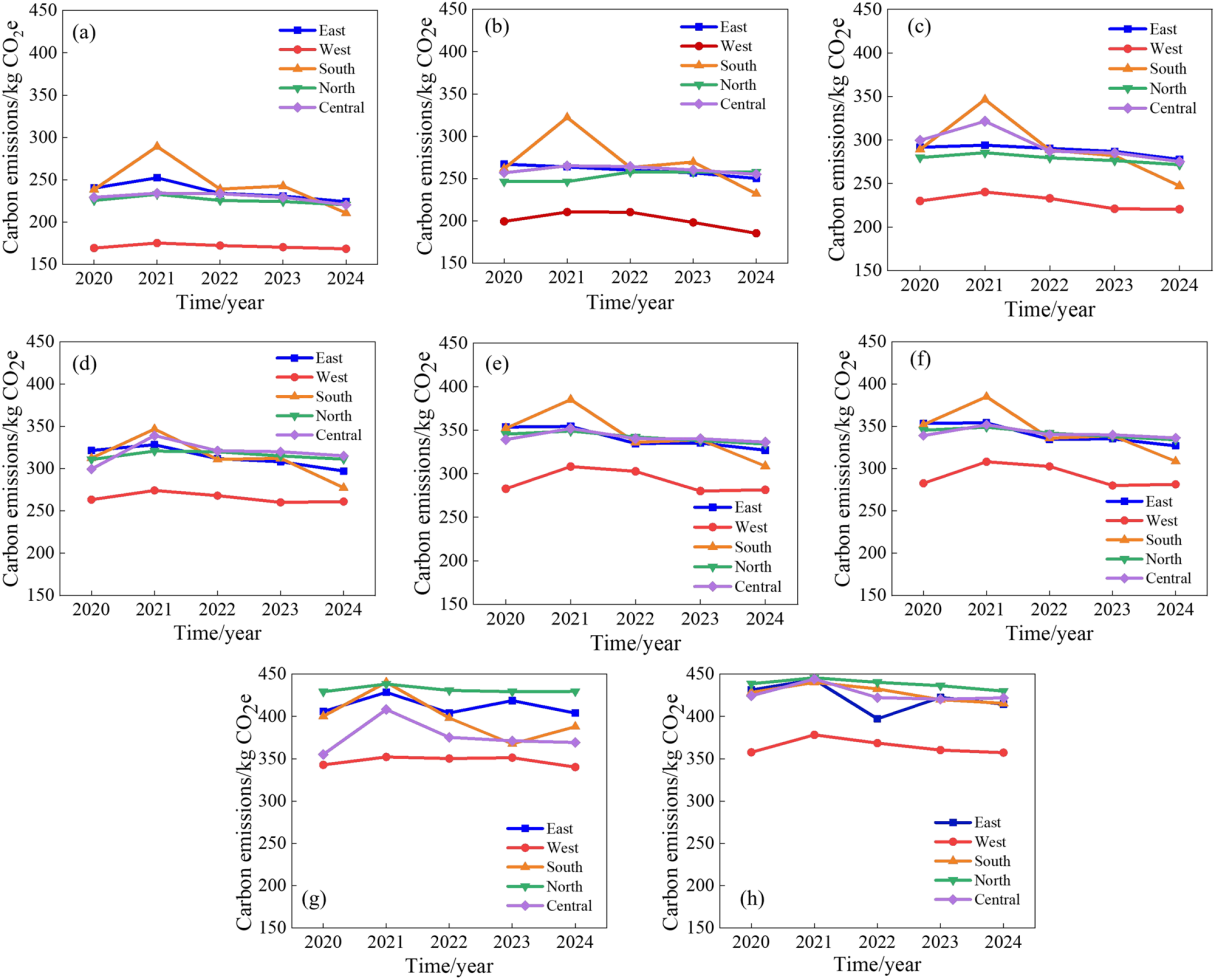
Temporal and spatial characteristics of carbon emissions in Shandong Province: (**a**–**h**) represents concrete strength grade C25-C60.

To illustrate the effect of economic, political, and cultural factors on carbon emissions over time in different regions, this study analyzed the temporal and spatial variations of concrete carbon emissions in Shandong Province over the past five years, as shown in Fig. 18. From a temporal perspective, the carbon emissions of concrete showed a slight downward trend (about 6%) between 2021 and 2024, which mainly benefited from the green building incentive policy to reconstruct the market demand. In 2021, the Shandong Provincial Government issued the Measures for Promoting Green Development of Urban and Rural Construction^47^, which emphasized the promotion of green buildings and green building materials. Subsequently, the 14th Five-Year Plan for Green Building and Building Energy Efficiency in Shandong Province^48^ further proposed improving the green building evaluation system by integrating green materials and energy efficiency requirements. Enterprises actively responded to policy needs and promoted the development of low-carbon concrete in the construction industry. On the other hand, China proposed the “dual carbon” goal in 2020, but specific emission reduction measures were gradually implemented after 2021, and a short-term lag effect may occur in this process.

In terms of spatial distribution, the concrete carbon emission intensity in Southern Shandong is the highest, approximately 15% higher than that of Western Shandong. This difference is primarily attributed to variations in regional industrial structure and the spatial differentiation of raw material supply chains^49,50^. As a traditional building materials industry cluster within Shandong Province, the southern region exhibits a higher cement dosage in concrete production than other areas. The fossil energy consumption in its cement production process directly pushes up the upstream raw material carbon emissions. At the same time, the aggregate transportation in this region mostly relies on road freight, while Western Shandong is close to the sand and gravel production area in Hebei and primarily utilizes railway or water transport, resulting in relatively low carbon emission intensity in the logistics sector. To enhance the broader applicability of the regional findings, we further compared the carbon emission factors obtained in this study with national and international authoritative datasets, including the Intergovernmental Panel on Climate Change (IPCC) Emission Factor Database (EFDB) and the Ecoinvent dataset. The results show that the emission factors for key materials such as cement, aggregates, and electricity in Shandong Province generally fall within the typical ranges reported by these datasets, while exhibiting notable regional differences. For example, the average cement-related emission factor in Shandong is slightly higher than the national average reported in EFDB, mainly due to differences in the regional electricity mix and relatively higher cement usage.

### Random model of carbon emission

**Fig. 19:**
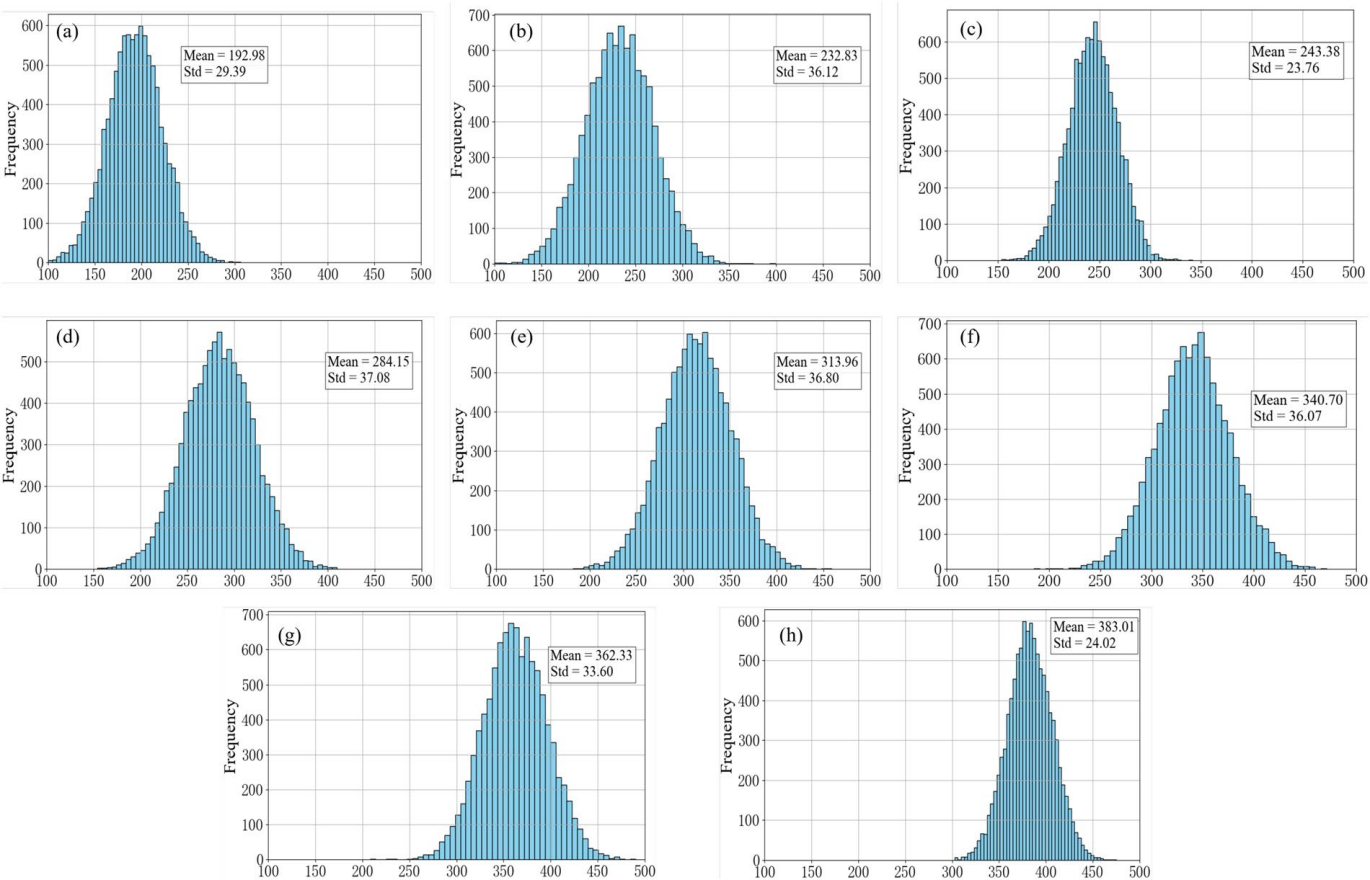
Probability Distribution of Concrete Carbon Emissions Based on Monte Carlo Simulation: (**a**–**h**) represents concrete strength grade C25-C60.

This study combines collected data with Monte Carlo simulation method to construct a random model of carbon emission calculation for concrete, which effectively overcomes the limitations of traditional theoretical models in quantifying uncertainty^51,52^. Based on the previous analysis of the statistical characteristics of various factors influencing concrete carbon emissions, this study further employed a Monte Carlo simulation with 10000 iterations for all random variables to establish a probability distribution model of carbon emissions for different strength grades of concrete, as shown in Fig. 19. The simulation results show that the carbon emission intensity shows an obvious nonlinear growth trend with the increase of concrete strength, and the carbon emission discreteness of high-strength grade concrete is larger (C25:192.98 ± 29.39 kgCO_2_/m^3^; C60: 383.01 ± 24.02 kgCO_2_/m^3^). The KS test (p > 0.05) confirmed that the carbon emission data of concrete of each strength grade obeyed the normal distribution, which was in line with the expectations of material science theory. The model not only evaluates the overall level of carbon emissions of concrete across different strength grades in Shandong Province, but also quantified its fluctuation range.

In order to verify the reliability of the stochastic model for carbon emission accounting of concrete, this study conducted a multidimensional comparison between the field survey data and the Monte Carlo simulation results, and established four key error evaluation indicators: mean absolute error (MAE = 4.82 kgCO_2_/m^3^), root mean square error (RMSE = 6.28 kgCO₂/m^3^), mean absolute percentage error (MAPE = 1.66%) and determination coefficient (R² = 0.9902). The verification results show that all error indicators have reached the ideal level (MAPE < 2%, R² > 0.99), proving that the model can accurately reflect the carbon emission characteristics of concrete of various strength grades.

### Sensitivity analysis of influencing factors

**Fig. 20:**
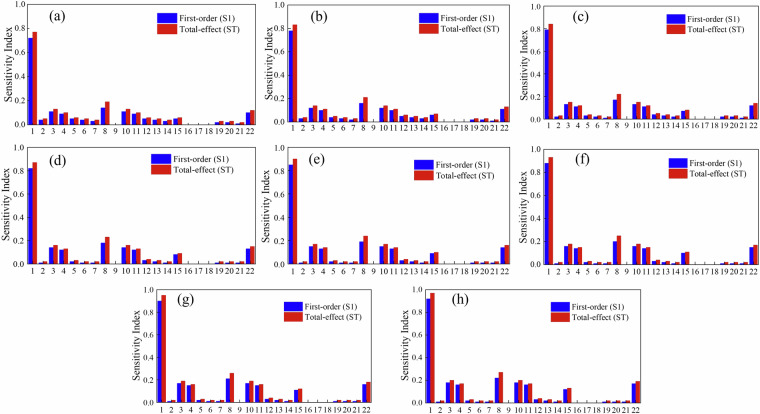
Comparison bar charts of the sensitivity indices of 22 influencing factors for concrete strength grades C25-C60. (1: cement consumption; 2: water consumption; 3: sand consumption; 4: gravel consumption; 5: fly ash consumption; 6: slag consumption; 7: chemical admixture consumption; 8: cement transportation distance; 9: water transportation distance; 10: sand transportation distance; 11: gravel transportation distance; 12: fly ash transportation distance; 13: slag transportation distance; 14: chemical admixture transportation distance; 15: cement transportation method; 16: water transportation method; 17: sand transportation method; 18: gravel transportation method; 19: fly ash transportation method; 20: slag transportation method; 21: chemical admixture transportation method; 22: electricity consumption).

Based on Variance-based Global Sensitivity Analysis (Sobol method), a quantitative evaluation of 22 uncertain factors affecting carbon emissions of concrete was conducted, as shown as Fig. 20. In detail, 1–7 represent the consumption of raw materials, 8–14 represent the transportation distances of raw materials, 15–21 represent the transportation methods of raw materials, and 22 represents electricity consumption. The sensitivity analysis indicates that, within the C25-C60 concrete strength range, cement dosage is the primary factor affecting carbon emissions. The first-order and total sensitivity indices (S1 and ST) increase linearly from 0.72/0.77 at C25 to 0.92/0.97 at C60 (R^2^ > 0.95), indicating that with each increase of one concrete strength grade, the contribution of cement to carbon emissions rises by approximately 5%. This finding is consistent with Zhang *et al*.^53^, who conducted a sensitivity analysis of life cycle carbon emissions in full-depth reclamation using Portland cement and found that variations in cement content have the most significant impact. When cement dosage changed by ±20%, carbon emissions varied by approximately ±17%, while the effects of other factors, such as energy consumption, were minimal (<2%). These results collectively confirm that cement dosage dominates concrete carbon emissions and represents a key leverage point for reducing the overall carbon footprint.

**Fig. 21:**
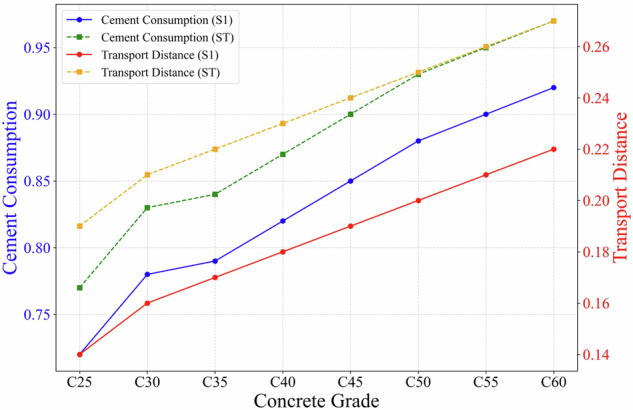
Curves of Sensitivity of Cement Usage and Transportation Distance with Strength Variation.

Meanwhile, the sensitivity index of cement dosages shows obvious strength dependence, as illustrated in Fig. 21. The S1 and ST indices increase linearly from 0.72/0.77 at C25 to 0.92/0.97 at C60 (R^2^ > 0.95). This trend indicates that with each increase of one concrete strength grade, the carbon emission contribution from cement dosage rises by approximately 5%. The sensitivity index of cement transportation distance shows a relatively stable trend with the change of concrete strength grade. The S1 strength grade fluctuates between 0.14–0.22, and the ST strength grade fluctuates between 0.19–0.27. The coefficient of variation (CV) is less than 15%, indicating that the impact of transportation distance on concrete of different strength grades is consistent. Therefore, the key to reducing carbon emissions from concrete lies in optimizing cement dosages and its supply chain logistics. Especially in high-strength concrete, balancing strength and carbon emissions to optimize both performance and environmental benefits is crucial.

## Discussion

This study systematically quantified the carbon footprint of ready-mixed concrete throughout its full production cycle based on field surveys at commercial concrete plants, providing a foundation for more accurate carbon accounting. The proposed methodology offers a robust framework that incorporates variability and uncertainty in raw material consumption, transportation, and electricity use, improving the accuracy of carbon footprint assessments. Probabilistic models developed for each strength grade refine conventional deterministic approaches, producing more representative results and enabling identification of emission-intensive stages. These findings not only support cross-regional comparisons but also provide actionable insights for policymakers and industry stakeholders. Effective strategies to reduce concrete emissions include optimizing cement content in concrete mixes, promoting supplementary cementitious materials such as fly ash or slag, improving energy efficiency in production processes, and optimizing logistics, for example by sourcing aggregates locally or adopting low-carbon transportation methods. By highlighting key emission drivers and potential interventions, this work can guide targeted mitigation strategies and inform region-specific carbon management policies. Furthermore, to enable continuous and fine-grained tracking of the spatiotemporal evolution of concrete-related carbon emissions. Such periodic updates would enhance the long-term applicability of the dataset and support region-specific carbon management and mitigation policies.

Optimizing concrete mixture design is among the most effective strategies for reducing emissions. Studies have shown that partial replacement of Portland cement with supplementary cementitious materials (SCMs), such as fly ash or slag, can significantly reduce CO_2_ emissions without compromising long-term strength^54,55^. Similarly, blending SCMs with limestone fillers can lower emissions by 20–40% while maintaining mechanical performance^2^. Process-level improvements, including optimized moisture control, automated batching, energy-efficient mixing, and enhanced curing, can further reduce energy consumption^56,57^. In addition, carbonation curing has been reported to influence both strength development and microstructure, providing further evidence for the potential of low-carbon curing strategies^58^. 

In addition to technical measures, our revised manuscript emphasizes that effective policy and regulatory frameworks are essential to accelerating low-carbon transitions in the concrete industry. Mandatory efficiency standards, updated specifications encouraging the use of SCMs and low-carbon materials, economic incentives, carbon pricing mechanisms, and strengthened reporting requirements for energy use and emissions can create enabling conditions for mitigation. Collaborative governance involving government agencies, concrete producers, equipment suppliers, and research institutions is also crucial to ensure that policies are both feasible and impactful. Based on our findings, we recommend establishing energy-efficiency benchmarks for concrete plants, providing incentives for equipment retrofits, and implementing pilot programs integrating digital process control and intelligent energy-management systems to guide industry-wide emission-reduction efforts.

### Limitations of the field survey data and carbon emission factor dataset for concrete in shandong province

Although great efforts were made to guarantee the reliability of this dataset, the potential uncertainties in the data collection process were still unavoidable, mainly due to the scope and time period of data collection. At the same time, although this study collected different raw material brands and manufacturers during the survey process, the impact on carbon emissions was not specifically reflected in the specific accounting of carbon emissions. The “National Carbon Emission Trading Market Covering Steel, Cement, and Aluminum Smelting Industry Work Plan” released this year pointed out that by the end of 2025, cement plants will successively announce the cement carbon emission factors of different manufacturers. On this basis, we will adjust the concrete carbon emission factors prepared by cement from different manufacturers obtained through the survey. Second, the data mainly come from field surveys and self-reported information by concrete plants, which may introduce potential biases. Third, the temporal coverage is limited, starting from 2020, and cannot reflect longer-term trends. Fourth, although the Monte Carlo simulation considered the probability distributions of raw materials and transportation factors, uncertainties inherent in the emission factors themselves were not included. Future research could incorporate publicly available cement emission factors and longer time-series data to further improve the concrete carbon footprint dataset. These shortcomings should be considered by users.

## Usage Note

This dataset is an important supplement to the carbon emission estimates of existing plant-level and provincial emission inventories in Shandong Province. The dataset covers five regions of Shandong Province. This dataset can be used to evaluate the impact of production capacity and equipment of different commercial concrete mixing stations in provinces and cities on energy consumption and CO_2_ emissions, which will help to achieve energy and environmental benefits. Managers and technicians can analyze this data to make informed decisions on concrete mix proportions, energy consumption, and supply chains. The dataset is open to the public.

## Date availability

Our dataset is stored on the website of Science Data Bank website. It is accessible through a link: 10.57760/sciencedb.28528^40^.

## Data Availability

This article uses Monte Carlo simulation to generate a stochastic model for carbon emissions accounting, and the corresponding code has been shared on GitHub and can be accessed via the following link: https://github.com/ZJ675980/Random-model-of-carbon-emission-accounting-for-concrete.git.
